# Advancing multimer analysis of von Willebrand factor by single-molecule AFM imaging

**DOI:** 10.1371/journal.pone.0210963

**Published:** 2019-01-15

**Authors:** Achim Löf, Gesa König, Sonja Schneppenheim, Reinhard Schneppenheim, Martin Benoit, Ulrich Budde, Jochen P. Müller, Maria A. Brehm

**Affiliations:** 1 Department of Physics and Center for NanoScience, LMU Munich, Munich, Germany; 2 Department of Pediatric Hematology and Oncology, University Medical Center Hamburg-Eppendorf, Hamburg, Germany; 3 MEDILYS Laborgesellschaft mbH, Hemostaseology, Asklepios Klinik Altona, Hamburg, Germany; National Cerebral and Cardiovascular Center, JAPAN

## Abstract

The formation of hemostatic plugs at sites of vascular injury crucially involves the multimeric glycoprotein von Willebrand factor (VWF). VWF multimers are linear chains of N-terminally linked dimers. The latter are formed from monomers via formation of the C-terminal disulfide bonds Cys2771-Cys2773’, Cys2773-Cys2771’, and Cys2811-Cys2811’. Mutations in VWF that impair multimerization can lead to subtype 2A of the bleeding disorder von Willebrand Disease (VWD). Commonly, the multimer size distribution of VWF is assessed by electrophoretic multimer analysis. Here, we present atomic force microscopy (AFM) imaging as a method to determine the size distribution of VWF variants by direct visualization at the single-molecule level. We first validated our approach by investigating recombinant wildtype VWF and a previously studied mutant (p.Cys1099Tyr) that impairs N-terminal multimerization. We obtained excellent quantitative agreement with results from earlier studies and with electrophoretic multimer analysis. We then imaged specific mutants that are known to exhibit disturbed C-terminal dimerization. For the mutants p.Cys2771Arg and p.Cys2773Arg, we found the majority of monomers (87 ± 5% and 73 ± 4%, respectively) not to be C-terminally dimerized. While these results confirm that Cys2771 and Cys2773 are crucial for dimerization, they additionally provide quantitative information on the mutants’ different abilities to form alternative C-terminal disulfides for residual dimerization. We further mutated Cys2811 to Ala and found that only 23 ± 3% of monomers are not C-terminally dimerized, indicating that Cys2811 is structurally less important for dimerization. Furthermore, for mutants p.Cys2771Arg, p.Cys2773Arg, and p.Cys2811Ala we found ‘even-numbered’ non-native multimers, i.e. multimers with monomers attached on both termini; a multimer species that cannot be distinguished from native multimers by conventional multimer analysis. Summarizing, we demonstrate that AFM imaging can provide unique insights into VWF processing defects at the single-molecule level that cannot be gained from established methods of multimer analysis.

## Introduction

**Fig 1:**
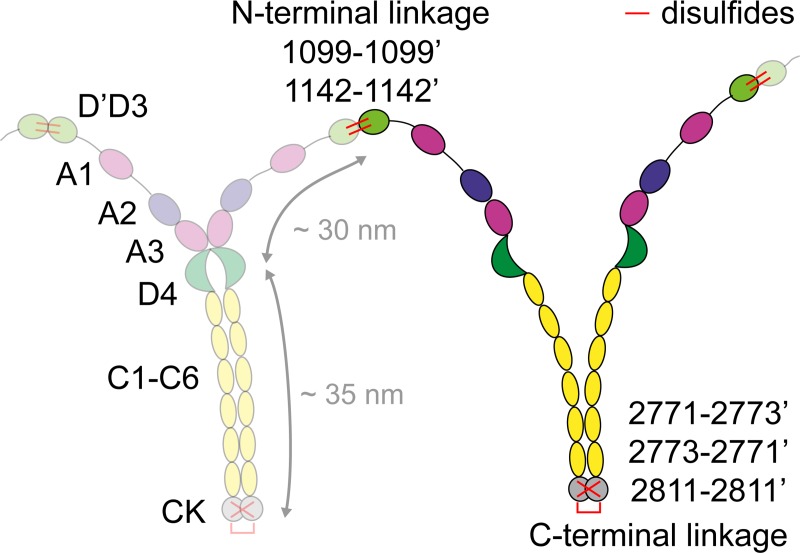
Schematic structure of VWF. Linear VWF multimers are built from dimers as smallest repeating subunits, which are linked in a head-to-head fashion via the disulfide bonds Cys1099-Cys1099’ and Cys1142-Cys1142’ between their N-terminal D`D3 assemblies. Dimers consist of two monomers–comprising the domains D`D3, A1, A2, A3, D4, C1-C6, and CK–that are linked in a tail-to-tail fashion via the disulfide bonds Cys2771-Cys2773’, Cys2773-Cys2771’, and Cys2811-Cys2811’ between their C-terminal CK domains.

The plasma glycoprotein von Willebrand factor (VWF) plays a crucial role in primary hemostasis [1–3]. Activated by increased hydrodynamic forces at sites of vascular injury [4], it recruits platelets to exposed subendothelial collagen and promotes the formation of platelet plugs. The hemostatically most active forms of VWF are its ultra-large multimers (UL-VWF), as they experience the highest forces and are capable of multivalent binding [1,5–7]. Mutations that alter the normal size distribution of VWF and result in reduction of large multimers can hence lead to the subtype 2A of von Willebrand Disease (VWD), the most common hereditary bleeding disorder [5,7]. Linear multimers are assembled in the trans-Golgi exclusively from dimers–VWF’s smallest repeating subunits–which are linked via the disulfide bonds Cys1099-Cys1099’ and Cys1142-Cys1142’ between their N-terminal D’D3 assemblies [8] (**Fig 1**). Multimers can partially be secreted constitutively or stored in Weibel Palade Bodies (WPBs) until release is stimulated by agonists [9,10]. In a recent study [11], which employed quantitative electrophoretic multimer analysis, fluorescence correlation spectroscopy, and total internal reflection fluorescence microscopy-based photobleaching, it was reported that the size distribution of VWF is exponential and may well result from a simple step-growth polymerization mechanism, where the number *N* of multimers of size *i (i* = 1 representing a dimer), is given by the expression *N*(*i*) = *N*_1_ × *p*^(*i-*1)^. Here, *N*_1_ is a constant fitting parameter representing the number of dimers after multimerization, and *p* describes the extent of multimerization. Larger values of *p* are indicative of samples in which large multimers are more abundant. For mutant p.Cys1099Tyr, which impairs N-terminal linkage of dimers, an exponential size distribution was still observed, albeit with a lower extent of multimerization.

VWF’s size distribution can also be affected by mutations in its C-terminal cystine-knot (CK) domain, as dimers are built in the endoplasmic reticulum (ER) from monomers that are linked in a tail-to-tail fashion via the three disulfide bonds Cys2771-Cys2773’, Cys2773-Cys2771’, and Cys2811-Cys2811’ between their CK domains by protein disulfide isomerase A1 (PDIA1) [12–15] (**Fig 1**). An impaired dimerization in the ER results in a more complex multimerization process than the above-mentioned simple step-growth multimerization. When dimerization is disturbed, multimers are assembled from dimers as well as from monomers, giving rise to the occurrence of ‘non-native’ multimers that comprise monomeric subunits [16,17]. Importantly, the N-terminal attachment of monomers to the ends of a multimer terminates its growth [5], as the resulting multimer ends on C-termini instead of N-termini. Thus, non-native multimers can be differentiated into ‘odd-numbered’ and ‘even-numbered’ ones, having a monomer attached to only one end and both ends, respectively. Odd-numbered multimers–i.e. multimers of sizes *i* = 1.5, 2.5, 3.5, and so forth–were, inter alia, reported for the VWD 2A/IID mutants p.Cys2771Arg and p.Cys2773Arg [14,16,17]. The relevance of the bond Cys2811-Cys2811’ was illustrated by the artificial mutation p.Cys2811Ala, which leads to the occurrence of odd-numbered multimers, albeit with an only minor reduction in the number and size of large multimers [13]. In line with the latter observation, this mutation has never been found in a patient with VWD [13]. As suggested by molecular dynamics (MD) simulations, the disulfide bond Cys2811-Cys2811’ was predicted not to be structurally essential for dimerization, but to shield and protect the disulfides Cys2771-Cys2773’ and Cys2773-Cys2771’ from reduction, thus rendering dimerization irreversible [13]. This hypothesis implies that in the absence of the protective disulfide bond partial reopening of the bonds Cys2771-Cys2773’ and Cys2773-Cys2771’ can occur.

Here, we employ single-molecule atomic force microscopy (AFM) imaging to directly visualize the size distribution of recombinant VWF multimer samples. The obtained results show excellent quantitative agreement with electrophoretic multimer analysis, which is the standard technique for multimer size distribution analysis and commonly used in diagnosis of VWD subtypes. Investigating several mutants that exhibit disturbed multimerization, we further demonstrate that AFM imaging can provide detailed insights into VWF processing and pathological defects at the single-molecule level that cannot be gained from electrophoretic multimer analysis alone. AFM imaging could hence aid conventional electrophoretic multimer analysis as a complementary technique and provide a valuable research tool to elucidate the pathological mechanisms of a variety of VWD-related VWF variants.

## Results

### AFM imaging enables quantitative analysis of VWF multimer size distributions

**Fig 2:**
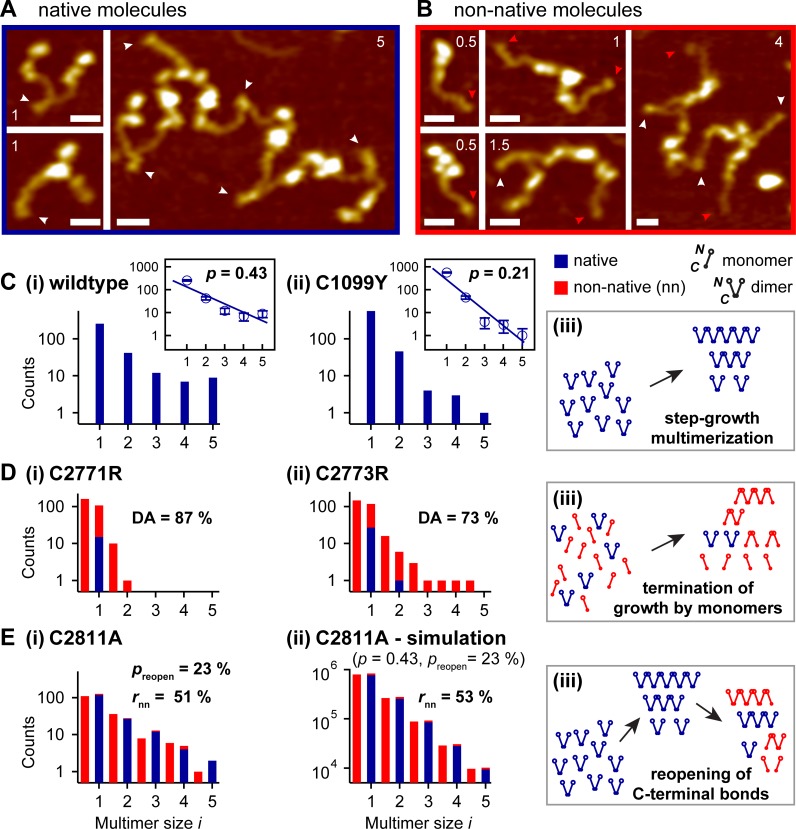
Multimer analysis of wildtype and mutant VWF by AFM-based imaging of individual molecules. (A, B) Representative AFM images of individual native (A) and non-native (B) VWF molecules. Numbers in images indicate the multimer size *i (i* = 1 corresponds to a dimer). White arrowheads mark paired, red arrowheads unpaired CK domains. For more details on the identification of dimeric and monomeric building blocks within VWF molecules, see **Materials and Methods** and **S1 Fig**. Scale bars represent 20 nm, range of color scale is 2.4 nm. (C) Size distributions of wtVWF (i) and mutant p.Cys1099Tyr (C1099Y, ii), and schematic of step-growth multimerization (iii). Insets in subpanels i and ii show linear fits to the data represented in logarithmic space, yielding values for the extent *p* of multimerization of 0.43 and 0.21, respectively. (D) Size distributions of VWF mutants p.Cys2771Arg (C2771A, i) and p.Cys2773Arg (C2773R, ii), and schematic of the underlying multimerization process (iii). Native and non-native molecules are depicted in blue and red, respectively. Non-native molecules are characterized by ending on a C-terminal CK domain (small, closed circle) at one or both termini, while native molecules end on N-terminal D’D3 assemblies (open circle). Monomers are “non-native” because they are never secreted after expression of wtVWF. From the observed size distributions, values for the dimerization abolishment of 87% and 73% were determined for p.Cys2771Arg and p.Cys2773Arg, respectively. (Ei) Size distribution of VWF mutant p.Cys2811Ala, for which non-native molecules had been hypothesized to result from reopening of disulfide-linked CK domains. The overall ratio of non-native molecules was found to be 51%. The (apparent) reopening probability was determined to be 23%. (Eii) Size distribution (shown for *i* ≤ 5) obtained from a simulation that assumed multimers to initially follow an exponential size distribution–with *p* = 0.43 as observed for wtVWF–and to afterwards reopen partially at their CK domains with the experimentally determined probability for p.Cys2811Ala. Simulations yielded, similarly to p.Cys2811Ala, a ratio of 53% non-native molecules, and very low fractions of even-numbered non-native molecules. (Eiii) Schematic representation of the hypothesized scenario of initial wildtype-like step-growth multimerization and subsequent reopening of C-terminal disulfide bonds within constituent dimers. The data presented in this figure are listed in **S1 Table**.

**Fig 3:**
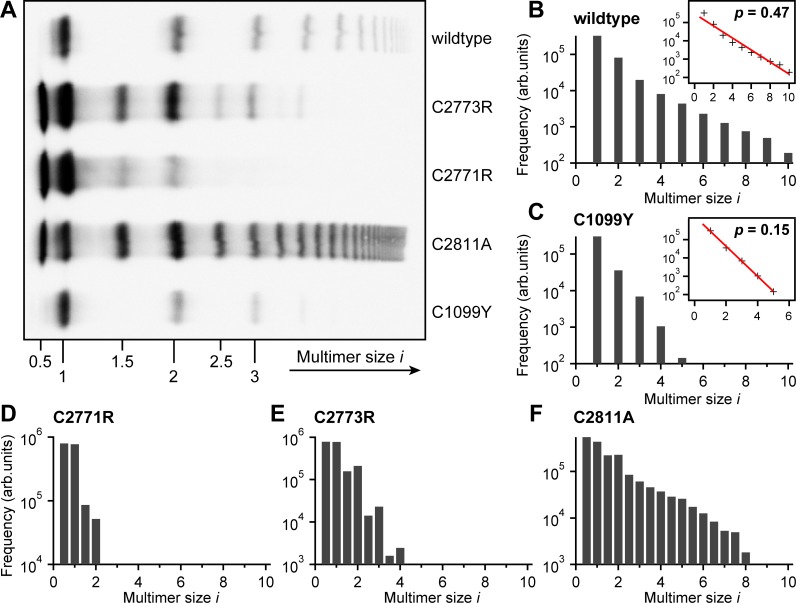
Quantitative electrophoretic multimer analysis of wildtype and mutant VWF. (A) High-resolution agarose gel of multimer samples. Numbers beneath bands indicate the multimer size *i (i* = 1 corresponds to a dimer). (B-F) Frequencies of molecules of size *i*, as determined from the gel (for more details, see **Materials and Methods** and **S4 Fig**), for wtVWF (B) and mutants p.Cys1099Tyr (C), p.Cys2771Arg (D), p.Cys2773Arg (E), and p.Cys2811Ala (F). Insets in panels B and C show linear fits to the frequency data represented in logarithmic space, yielding values for the extent *p* of multimerization of 0.47 and 0.15 for wtVWF and mutant p.Cys1099Tyr, respectively.

We first performed proof-of-principle AFM single-molecule imaging experiments with recombinant wildtype (wt)VWF and compared the results with those obtained by electrophoretic multimer analysis. To this end, wtVWF multimers were adsorbed onto a functionalized mica substrate (for details of sample preparation, see **Materials and Methods**) and imaged employing AFM (**Fig 2A and 2B and S1 Fig**). We directly determined the size distribution by counting the number *N* of molecules of multimer size *i*, where *i* = 1 denotes a dimer, *i* = 2 a tetramer, and so forth. We observed a distribution of multimer sizes that decayed in an approximately exponential fashion (**Fig 2Ci**), in line with a recent study [11]. Due to the exponential decay and the chosen single-molecule approach, multimers of sizes *i* > 5 were observed only occasionally in AFM images (**S1 Table**). We estimated the extent *p* of multimerization by fitting the expression *N*(*i*) = *N*_1_ × *p*^(*i*-1)^ to our data for multimer sizes up to *i* = 5, and obtained a value of 0.43 ± 0.11 (± 1 SD). This value is in excellent agreement with the value of 0.47 ± 0.03 that we obtained by quantitative luminescence intensity profiling of wtVWF electrophoretic multimer analyses (**Fig 3A and 3B**).

To test the capability of our AFM imaging approach to detect alterations in the size distribution, we further investigated the mutant p.Cys1099Tyr, which exhibits a well-described defect in multimerization [8,11]. This mutation, which impairs the N-terminal linkage of dimers, had been shown to also result in an exponential size distribution, but with a steeper decay than observed for wtVWF, corresponding to a lower extent of multimerization [11]. We indeed observed an exponential size distribution shifted towards smaller values of *i* compared with wtVWF (**Fig 2Cii**). We obtained a significantly lower value for *p* of 0.21 ± 0.07, again in good agreement with quantitative electrophoretic multimer analysis, which yielded *p* = 0.15 ± 0.01 (**Fig 3C**). These data show that AFM imaging is an adequate method to determine the degree of VWF multimerization in a quantitative fashion.

### Insights into VWF processing defects beyond conventional multimer analysis

#### Cys2771 and Cys2773 are crucial for VWF dimerization

It has previously been reported that the mutants p.Cys2771Arg and p.Cys2773Arg exhibit disturbed dimerization [14,16,17]. Electrophoretic multimer analysis revealed that both of these mutations lead to the formation of multimers containing odd numbers of monomers (‘odd-numbered multimers’) and to a deficit of large multimers (**Fig 3D and 3E**). While p.Cys2771Arg almost exclusively showed monomers and dimers, for p.Cys2773Arg multimers with sizes of up to *i* = 4 were observed, including also odd-numbered molecules (i.e. molecules with sizes of *i* = 0.5, 1.5, 2.5, and 3.5; **Fig 3D and 3E**). These data confirm the ability of p.Cys2773Arg to still form small multimers. We assessed the multimer size distribution of p.Cys2771Arg (**Fig 2Di**) and p.Cys2773Arg (**Fig 2Dii**) by AFM imaging and determined the number of both dimeric and monomeric building blocks incorporated into all observed VWF molecules. The obtained size distributions were again in excellent agreement with those obtained by conventional, electrophoretic multimer analysis. For the mutant p.Cys2771Arg, we found 96 ± 6% monomers (*i* = 0.5) and dimers (*i* = 1), and only rarely trimers (*i* = 1.5) and tetramers (*i* = 2). The mutant p.Cys2773Arg exhibited all the multimer sizes that were observed in the electrophoretic multimer analysis. However, in AFM imaging, the observed maximum multimer size was *i* = 4.5, i.e. one unit larger than in electrophoretic multimer analysis. This finding shows that AFM imaging, as a single-molecule technique, is in principle capable of detecting rare multimer species, which–due to the luminescence intensity detection limit–are not visible in electrophoretic multimer analysis.

A tremendous advantage of AFM imaging over electrophoretic multimer analysis is the possibility to distinguish native multimers from non-native even-numbered (*i* = 1, 2, 3, and so forth) multimers of the same size, i.e., from multimers that exhibit a monomeric building block at both ends (**Fig 2A and 2B, Diii**). For instance, AFM imaging can resolve the structural difference between C- and N-terminally linked dimers, while in electrophoretic analysis C- and N-terminally linked dimers are hidden in the same band. In other words, AFM imaging allows for counting non-native dimers and multimers individually. Our analysis showed that for p.Cys2771Arg, only 14 ± 4% of the observed dimers are native, i.e. linked C-terminally (**Fig 2Di**, note the logarithmic scale). For the mutant p.Cys2773Arg, this ratio was found to be 23 ± 5%. The observed difference between the two mutants goes hand in hand with the previous observation that the latter mutant is still able to form small multimers [13,17], as more native dimers are available for multimer formation.

We further quantified the degree of dimerization abolishment (DA)–i.e. the fraction of all monomers that are not C-terminally dimerized (*cf*. **Methods**)–and found DA values of 87 ± 5% and 73 ± 4% for p.Cys2771Arg and p.Cys2773Arg, respectively. Taken together, these data confirm that both cysteines, Cys2771 and Cys2773, are essential for dimerization. Cys2771 appears to be slightly more important, in the sense that the investigated mutation of this cysteine results in a more pronounced loss of dimerization than the mutation of Cys2773, indicating that the absence of Cys2771 cannot be compensated significantly by alternative disulfide bonds. At least partial compensation is however possible in the absence of Cys2773.

In addition, it is noteworthy that our data indicate an approximately equal probability for the N-terminal attachment of dimers and monomers to an assembling VWF molecule during multimerization. We separately determined the fractions of dimeric and monomeric building blocks N-terminally linked to at least one other building block and observed no significant difference between these fractions for p.Cys2771Arg (42 ± 13% and 55 ± 4%, respectively), or for p.Cys2773Arg (60 ± 10% and 60 ± 4%, respectively).

#### Cys2811 plays a secondary role in VWF dimerization

To also investigate the impact of loss of the third cysteine involved in dimerization, Cys2811, we imaged the mutant p.Cys2811Ala, which is unable to form disulfide bond Cys2811-Cys2811’ [13] (**Fig 2Ei**). Approximately one-half of all molecules were observed to be non-native (rate of non-native molecules: *r*_nn_ = 51 ± 4%), and 33 ± 3% of all molecules were isolated monomers. The overall size distribution, however, exhibited an only minor shift towards smaller values of *i* compared with wtVWF, in line with observations from electrophoretic multimer analysis (**Fig 3F**). AFM imaging further revealed a fraction of 93 ± 9% native dimers and only 7 ± 2% N-terminally linked, non-native dimers (**Fig 2Ei**), showing that disulfide bond Cys2811-Cys2811’ is not crucial for formation of native dimers. Also for larger even-numbered multimers, i.e. for tetramers (*i* = 2), hexamers (*i* = 3) and so forth, only a very low fraction of non-native molecules was observed. These findings suggest that Cys2811 is structurally less important than Cys2771 and Cys2773 and plays a secondary role in dimerization.

The observed size distribution can be explained by a scenario in which mutation of Cys2811 directly, but only moderately, impairs dimerization via disulfide bonds Cys2771-Cys2773’ and Cys2773-Cys2771’ in the ER, e.g. by inducing structural changes in the CK domain. In this scenario, multimers would be assembled from both dimeric and monomeric building blocks, entirely analogous to the mechanism of mutants p.Cys2771Arg and p.Cys2773Arg described above, but with a markedly lower degree of DA of 23 ± 3%.

However, based on MD simulations, it has recently been postulated that disulfide bond Cys2811-Cys2811’ is not structurally essential for dimerization, but rather serves to shield and protect the disulfides Cys2771-Cys2773’ and Cys2773-Cys2771’ from reduction, thus rendering dimerization irreversible [13]. This hypothesis implies that in the absence of the protective disulfide bond Cys2811-Cys2811’ the other two bonds (Cys2771-Cys2773’ and Cys2773-Cys2771’) may undergo partial reopening. To test compatibility of our data with the hypothesis that reopening of C-terminal disulfide bonds can occur, we assumed an extreme scenario in which initially wildtype-like formation of exclusively native, i.e. C-terminally linked, dimers takes place, which would then undergo normal multimerization. After multimerization, multimers would break up randomly between CK-domains of constituent monomers, yielding non-native multimers (**Fig 2Eiii**). In this scenario, all apparent monomeric building blocks observed in our measurements would have originated from the reopening of dimeric building blocks. Under this assumption, we determined the probability *p*_reopen_ for the reopening of a dimeric building block from our data, and obtained a value of *p*_reopen_ = 23 ± 3%. We then performed simple simulations *in silico* (**Fig 2Eii**). To this end, we constructed an initial exponential size distribution of multimers with an extent of multimerization of *p* = 0.43, i.e. with the value that was obtained experimentally for wtVWF, and with a large *N*_1_ of 10^6^. In the next step, each C-terminal dimerization site within multimers, i.e. each pair of disulfide-linked CK domains, was broken with an equal probability of *p*_reopen_ = 23%. The resulting size distribution very closely matched the experimentally observed one. In particular, we found the overall ratio of non-native molecules to be *r*_nn_ = 53%, agreeing excellently with the ratio of 51% that was determined experimentally. Moreover, the fractions of even-numbered non-native molecules were closely reproduced, e.g., 8% non-native dimers, compared with 7% found in the experiment. Our simulations thus show that the experimental data obtained in the absence of disulfide bond Cys2811-Cys2811’ are compatible with the proposed scenario involving reopening of C-terminal disulfide bonds after dimerization. It should further be noted that also a situation combining the two different scenarios described above, i.e. a situation in which dimerization of monomers and reopening of dimers occur in parallel already in the ER, could result in the same final size distribution after multimerization. Thus, definite discrimination between these possibilities will likely require elaborate *in vivo* measurements and is beyond the scope of this study.

**Fig 4:**
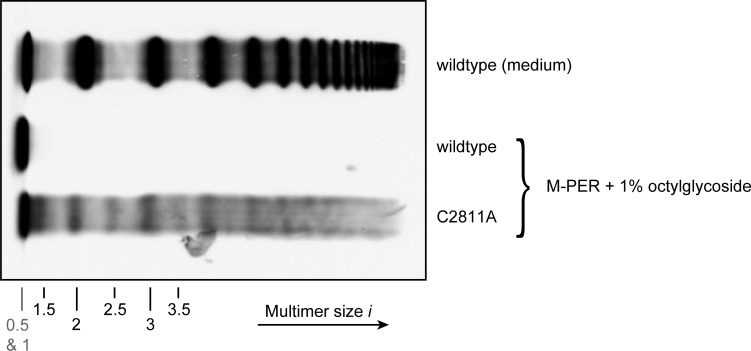
Stability of pseudo-WPBs in HEK293 cells expressing wtVWF or mutant p.Cys2811Ala. HEK293 cells stably overexpressing wtVWF (middle lane) or mutant p.Cys2811Ala (bottom lane) were lysed using M-PER lysis buffer containing 1% (w/v) octylglycoside. The lysates were analyzed by electrophoretic multimer analysis. As size reference, wtVWF secreted into the medium is shown (top lane).

A third scenario, in which C-terminal degradation occurs extracellularly, could be excluded by cell lysis experiments. To investigate if odd-numbered multimers already exist intracellularly, HEK293 cells stably overexpressing wtVWF or mutant p.Cys2811Ala were lysed using M-PER lysis buffer containing 1% (w/v) octylglycoside **(Fig 4)**. For cells expressing mutant p.Cys2811Ala, this treatment resulted in lysis of pseudo-Weibel-Palade bodies (pseudo-WPBs), and the cell lysate exhibited all multimer sizes, including non-native multimers, as seen in electrophoretic multimer analysis (bottom lane). Cell lysate of cells expressing wtVWF did not exhibit multimers (middle lane), indicating that pseudo-WPBs containing wtVWF cannot be disrupted under identical conditions.

## Discussion

In this study, we introduced the use of single-molecule AFM imaging as a research tool to characterize the size distribution of VWF multimers. We used recombinant VWF expressed in HEK293 cells, which do not intrinsically possess WPBs, but form pseudo-WPBs induced by expression of VWF. Although WPBs and pseudo-WPBs most likely are not identical in their composition, HEK293 cells have been shown to produce multimer size distributions that very closely match those found in plasma samples [18], and therefore are a commonly used cell line for recombinant VWF production (e.g.: [19–21]). The usage of recombinant protein has a couple of advantages: 1) VWF is secreted into the serum-free medium of choice, making complex purification protocols unnecessary. 2) Absence of ADAMTS13 provides uncleaved VWF samples. 3) Patients are not burdened with voluminous blood draws, which would be necessary for complex purification protocols to yield plasma VWF of high enough purity for imaging. 4) The expression in HEK293 cells reproduces the homozygous state. Although it is clinically much less common than the heterozygous state, it can be analyzed more straight-forwardly and helped us to gain deeper insights into how the different mutations disturb VWF processing. 5) If necessary, reproduction of the heterozygous state is also possible by co-expression of wildtype and variant VWF, and good agreement between the size distribution of heterozygous recombinant multimers and respective patient plasma samples has been reported [18].

Thus, while we do not see immediate application of our technique as a diagnostic tool for analysis of patient samples, AFM imaging is an excellent research tool to better comprehend the processing defects of VWD-related VWF variants and the molecular mechanisms underlying certain VWD subtypes.

We demonstrated the utility of this AFM imaging approach by showing that it can provide quantitative insights into VWF processing defects that go beyond the results of conventional multimer analysis. To verify the validity of our imaging approach, we first investigated wtVWF and the mutant p.Cys1099Tyr, which impairs N-terminal disulfide-linkage between dimers [8,11]. The results obtained from AFM imaging were in very good agreement with data from quantitative electrophoretic multimer analysis and fully consistent with results from an earlier study that had employed a combination of quantitative electrophoretic multimer analysis, fluorescence correlation spectroscopy, and total internal reflection fluorescence microscopy-based photobleaching for investigating multimer size distributions [11]. These findings show consistency between AFM imaging and the established method of multimer analysis by electrophoresis and prove the capability of AFM imaging to detect multimerization defects of VWF. It should however be pointed out that a quantitative multimer analysis by AFM imaging is limited to rather small multimer sizes due to two different aspects. First,–much like for established methods of multimer analysis–the exponentially decaying size distribution of VWF limits the throughput at high multimer sizes. Since large multimers are much less frequent than smaller ones, reliable statistics can, with reasonable effort, only be collected from AFM images for smaller multimers (*i* ≤ 5 in this study). However, larger multimers are still fairly commonly observed (**S1 Table**; a multimer of size *i* = 12 is highlighted in **S3 Fig**), which would allow for detection of structural anomalies that might potentiate in larger multimers. For variant p.Cys2811Ala, for instance, nine molecules with sizes of *i* > 5 were observed, of which four had sizes of *i* > 10. Also in these larger multimers we were able to identify processing defects and to distinguish native (five) from non-native (four) multimers (**S1 Table**). Second, the unambiguous identification of a multimer’s dimeric or monomeric subunits becomes increasingly complicated for increasing multimer size due to possible self-overlaps or close colocalization with other molecules.

It is worth noting that the exponential size distributions observed both for wtVWF and mutant p.Cys1099Tyr are fully in line with a step-growth multimerization process with dimers as sole building blocks (**Fig 2Ciii**). In contrast, for the mutants p.Cys2771Arg and p.Cys2773Arg, which lead to a marked dimerization abolishment [12,13,16,17], multimers are assembled from two different building blocks–dimeric and monomeric ones–by N-terminal linkage [14], implying a compromised multimerization process: the growth of a multimer is terminated by the attachment of monomers via the D’D3 assembly [5], resulting in the formation of non-native multimers and a severe lack of large multimers (**Fig 2Diii**).

The direct visualization of multimeric VWF samples by AFM imaging proved to be a powerful approach for multimer analysis, complementary to the established method of SDS-agarose gel electrophoresis, where after size separation and blotting multimer bands are typically detected using anti-VWF antibodies and visualization by luminescence [22,23]. As AFM imaging can directly visualize monomeric and dimeric building blocks of VWF, both individually and as constituents of multimers, the imaging approach that we chose in this study is capable of providing additional and quantitative information that cannot be gained from electrophoretic analysis. In particular, AFM imaging does not only allow for identifying odd-numbered non-native molecules, but also for distinguishing between native and even-numbered non-native molecules of the same size *i*, for instance between dimers that are linked C-terminally and N-terminally, respectively. The latter discrimination is not directly possible by electrophoretic multimer analysis, as native and non-native molecules of the same size *i* are located in the same band in a gel. In principle, information about N- or C-terminal linkage could be obtained by preceding proteolysis of mutant VWF using ADAMTS13, which would result in different proteolytic fragments. However, these fragments would again have to be analyzed by Western blotting, making quantitative analysis difficult. In contrast, AFM imaging enabled us to directly quantify how many native dimers can still be formed by the mutants p.Cys2771Arg, p.Cys2773Arg, and p.Cys2811Ala. The data we obtained confirm the severe inhibiting effects on dimerization–and consequently multimerization–of the first two mutations, and show that mutation p.Cys2771Arg affects dimerization more severely than p.Cys2773Arg (**Figs 2D, 3D and 3E** and [14]). The fact that 13% and 27% of all monomers are still C-terminally dimerized for p.Cys2771Arg and p.Cys2773Arg, respectively, suggests that occasionally alternative disulfide bonds such as Cys2773-Cys2773’ or Cys2771-Cys2771’ can be formed [13,14]. The lower rate of C-terminal dimerization of p.Cys2771Arg compared with p.Cys2773Arg may be explained by a higher degree of structural change within the CK domain in case of a mutation of Cys2771 than for a mutation of Cys2773. This assumption is supported by previously published structure modeling and MD simulations [14].

Whereas Cys2771 and Cys2773 were confirmed to be crucial for C-terminal dimerization, significant formation of C-terminally linked, native dimers can still occur in the absence of Cys2811, as observed for mutant p.Cys2811Ala. Consequently, an only minor overall shift to smaller values of *i* compared with wtVWF was found for this mutant. The large fraction of C-terminally linked monomers (77%; DA = 23%) indicates that mutation p.Cys2811Ala does not severely inhibit the formation of the dimerizing bonds Cys2771-Cys2773’ and Cys2773-Cys2771’, indicating that Cys2811 is structurally less important for dimerization, and suggesting a different role for disulfide bond Cys2811-Cys2811’. For instance, it might render dimerization irreversible by shielding the other two disulfides from reduction [13]. As corroborated by simple simulations, our data on p.Cys2811Ala are consistent with such a scenario in which the absence of disulfide bond Cys2811-Cys2811’ still allows normal dimerization via disulfide bonds Cys2771-Cys2773’ and Cys2773-Cys2771’, but results in subsequent partial reopening of these bonds (**Fig 2Eiii**). It appears plausible that such reopening might already occur in the ER, where VWF monomers are dimerized by PDIA1 [13], an enzyme that is not only able to form but also to reduce disulfide bonds. In other words, lack of disulfide bond Cys2811-Cys2811’ would, in this scenario, shift the equilibrium towards increased reduction of disulfides Cys2771-Cys2773’ and Cys2773-Cys2771’. Reopening might potentially also occur at the acidic pH values encountered during tubulation of VWF [6,24], as disulfide reduction has been shown to be possible at the acidic pH of endosomes [25] (pH = 5–5.5) [26]. Since in cell lysis experiments we observed pseudo-WPBs containing p.Cys2811Ala to be less stable than pseudo-WPBs containing wtVWF, it might be possible that Cys2771-Cys2773’ and Cys2773-Cys2771’ are reduced during tubulation, leading to a less compact packing. However, as described before, the size distribution observed for mutant p.Cys2811Ala could also result from a scenario in which the lack of Cys2811 directly, but only moderately, impairs formation of disulfide bonds Cys2771-Cys2773’ and Cys2773-Cys2771’–e.g. due to slight structural changes within the CK domain induced by mutation of Cys2811–, without reopening of formed disulfides. Discrimination between these possibilities may be facilitated in the future by more sophisticated *in vivo* measurements or by new insights from structural studies or MD simulations.

## Conclusions

Put succinctly, our data on selected VWF mutants are fully in line with the recently proposed picture of VWF dimerization: Cys2771 and Cys2773 are the essential starting points of dimerization and bonds Cys2771-Cys2773’ and Cys2773-Cys2771’ are the structural bonds that connect CK domains of monomers. A loss of Cys2771 has a more severe effect than a loss of Cys2773, indicating that an alternative bond Cys2773-Cys2773’ is formed less readily than Cys2771-Cys2771’. Cys2811 in contrast appears to play a secondary role in dimerization, as loss of Cys2811 still allows for significant formation of native dimers and consequently larger multimers. From a more methodological point of view, we showed that AFM imaging is a powerful approach to assess the size distribution of VWF, which can help to gain a quantitative understanding of the processes involved in VWF’s multimerization and in multimerization defects at the single-molecule level. In particular, direct visualization of individual molecules by AFM imaging enables detection of structurally anomalous molecules, even if they are indistinguishable from native molecules in electrophoretic multimer analysis. In the future, AFM imaging may therefore aid electrophoretic multimer analysis as a complementary method to better comprehend the pathological mechanisms of elusive VWF variants, especially in cases where subtle structural differences are expected to play a role.

## Materials and methods

### Cell culture and VWF expression in HEK293 cells

Wildtype (wt) VWF was expressed using the full-length cDNA of human wtVWF within the mammalian expression vector pcDNA3.1 [27]. Single missense mutations p.Cys1099Tyr, p.Cys2771Arg, p.Cys2773Arg and p.Cys2811Ala were inserted into the same vector by site-directed mutagenesis employing the QuikChange kit (Stratagene) and the resulting vectors were sequenced to confirm insertion of the mutations and absence of any additional unwanted mutations. Expression yields untagged VWF variants. Primer sequences are available upon request. Top10 supercompetent cells (Invitrogen) were transformed with these vectors and plasmid purification was performed using the Endofree Plasmid Maxi Kit (QIAGEN). 4 μg vector DNA were used to transfect HEK293 cells (2*10^6^) employing Lipofectamine 2000 (Thermo Fisher Scientific) according to the manufacturer’s instructions. The cells were selected for stable expression for 2 weeks by adding 500 μg/ml G418 (Thermo Fisher Scientific) to the Dulbecco modified Eagle medium (Thermo Fisher Scientific) with 10% [vol/vol] fetal bovine serum. Since VWF is secreted by the cells, 72 hours before harvesting the VWF-containing medium, the medium was exchanged with serum-free OPTIPRO-SFM medium (Thermo Fisher Scientific).

### AFM imaging

Recombinant VWF constructs in serum-free medium were purified and buffer exchanged by repeated centrifuge filtration using Amicon Ultra-15 NMWL 100 kDa (Merck Chemicals, Darmstadt, Germany), using buffer adjusted to pH 7.4, containing 20 mM Hepes, 150 mM NaCl, 1 mM CaCl_2_, and 1 mM MgCl_2_. No further steps of purification were performed. We refrained from using VWF constructs equipped with N- or C-terminal peptide tags for high-affinity purification, as such tags might influence VWF’s multimerization. We further refrained from purifying our untagged samples via a VWF affinity matrix, as we observed such purification procedures to markedly affect the conformation of VWF’s N-terminal portions (**S2 Fig**), possibly due to the required relatively harsh elution conditions.

Substrates for AFM imaging were prepared as previously described [28,29]. In brief, 20 μl of ≈3 μg/ml of VWF, which is overall negatively charged, were adsorbed onto a mica substrate coated with positively charged poly-L-lysine. After 30 s, slides were gently rinsed with ultrapure water and dried in a stream of nitrogen. AFM images of 2 μm × 2 μm and 1024 × 1024 pixels were recorded in tapping mode in air, using an MFP-3D AFM (Asylum Research, Santa Barbara, CA) and cantilevers with silicon tips (AC160TS, Olympus, Japan), possessing a nominal spring constant of 26 N/m and a resonance frequency of approximately 300 kHz. Raw image data were processed using SPIP software (v6.4.4; Image Metrology, Denmark). Image processing involved plane correction (third order polynomial plane-fitting), line-wise flattening according to the histogram alignment routine, and Gaussian filtering. An exemplary full image for wtVWF is shown in **S3 Fig**.

Images were analyzed by directly counting VWF molecules and determining the number of dimeric and monomeric subunits for each molecule. Poisson noise (1 SD) was assumed to estimate statistical uncertainties in counting. Exemplary images of individual VWF molecules are shown in **Fig 2A and 2B**. Reliable identification of VWF molecules and their subunits was enabled by their large size and their characteristic structure (**S1 Fig**): each monomer possesses a higher “head” portion with a typical length of approximately 30 nm, comprising the larger N-terminal domains, and a rather uniform “tail” region of markedly lower height with a length of approximately 35 nm, comprising the C-terminal domains C1-C6 and CK [28–30] (*cf*. **Fig 1**). The CK domain of a monomer typically exhibits a slightly larger height than the rest of the “tail”. Paired CK domains within a dimer show a markedly increased height compared to CK domains in individual monomers (**S1 Fig**). Thus, linear VWF multimers exhibit a characteristic alternating pattern of larger and higher, and thinner and lower portions. It should be noted that roughly half of the native dimers within a multimer adopt a rather compact conformation with their C-terminal “tails” zipped up into stems [28,29]. Occasional VWF molecules that could not be analyzed unambiguously, e.g. owing to colocalization with other VWF or contaminant molecules, were discarded from analysis.

### Calculation of dimerization abolishment

Defining the dimerization abolishment (DA) as the overall fraction of monomers–both isolated and as constituents of multimers–that are not C-terminally linked to another monomer, the DA is obtained by dividing the number of monomeric building blocks incorporated into all observed VWF molecules (including isolated monomers) by the sum of monomeric building blocks and two times the number of dimeric building blocks.

### Electrophoretic multimer analysis

Electrophoretic multimer analysis was performed as described [22,23,31]. In brief, VWF multimers were separated by SDS-agarose electrophoresis, transferred onto a nitrocellulose membrane, detected with anti-human VWF antibody-HRP linked (DAKO) and visualized by luminescence.

Frequencies of multimers of size *i* were extracted quantitatively from each blot lane by analyzing the luminescence intensity profile along a cross-section through the lane (**S4 Fig**). Intensity profiles exhibited clear peaks at the positions of bands visible in the blot, and were fitted with a multi-Gaussian function. Areas of fitted Gauss peaks were normalized by the respective multimer size *i* in order to obtain relative frequencies.

### Cell lysis

HEK293 cells stably overexpressing wtVWF or mutant p.Cys2811Ala were lysed by using M-PER (Thermo Fisher Scientific) including 1% (w/v) octylglycoside (Sigma) for 1 h at RT, in the presence of protease inhibitor mix (Roche).

## Supporting information

S1 Fig Analysis of VWF molecules in AFM images. (A) Identification of dimeric and monomeric building blocks within VWF molecules. Left: Exemplary image of an individual non-native VWF molecule of size *i* = 1.5, i.e. consisting of a dimer and an N-terminally attached monomer, with a schematic representation of the structure below. Scale bar represents 20 nm, range of color scale is 2.4 nm. Right: Height trace along the contour of the molecule (bottom), obtained by tracing the molecule from end to end, following local maxima in height (top). A clear height difference between lower C-terminal portions (C domains, yellow) and higher N-terminal domains is clearly visible. Due to the attachment of a single monomer, the molecule shown here ends on an N- and on a C-terminus, which exhibit markedly different heights. White arrowheads mark the paired–i.e. dimerized–CK domains of the dimeric building block, and red arrowheads mark the unpaired CK domain of the monomeric building block. Again, a clear height difference is observed. It should be noted that height tracing of molecules was, for the vast majority of analyzed molecules, not necessary for reliable identification of their dimeric and monomeric building blocks, as the characteristic pattern of alternating higher and lower portions of VWF is also directly visible from the color scale. (B) Further exemplary images of VWF multimers with schematic structures shown below for illustration. Left: native molecule of size *i* = 5, i.e. consisting of five dimers and thus ending on two N-termini. Right: non-native molecule of size *i* = 4, i.e. consisting of three dimers and two N-terminally attached monomers, thus ending on two C-termini. White and red arrowheads mark paired and unpaired CK domains, respectively. Scale bars represent 20 nm, range of color scale is 2.4 nm.
(EPS)

S2 Fig Effect of anti-VWF purification on VWF’s conformation. Dimeric VWF samples with a deletion of the pro-peptide (domains D1-D2, aa 26–763) were purified by different methods and afterwards buffer exchanged to buffer adjusted to pH 7.4, containing 20 mM Hepes, 150 mM NaCl, 1 mM CaCl_2_, and 1 mM MgCl_2_. (A) Dimers equipped with an N-terminal Strep-tag II [32] were purified via a StrepTactin column (HiTrap StrepTrap, GE Healthcare) and eluted at pH 7.4 using buffer containing 2.5 mM d-Desthiobiotin. AFM imaging of the purified sample (right) revealed conformations of dimers indistinguishable from those observed for unpurified samples (compare Fig 2A in the main text and Müller et al. [28]). The distribution of the maximum height observed for each individual VWF dimer (left) exhibits a narrow peak at approximately 2.5 nm. (B) Dimers possessing no peptide tags were purified using a Capture Select VWF affinity matrix (Life Technologies) and eluted at neutral pH using buffer containing 3 M sodium thiocyanate. Dimers purified by this procedure exhibited a marked change in the conformation of their N-terminal “head” domains. The latter appeared to clump together, resulting in a broadening as well as a shift of the distribution of the maximum height of individual dimers to larger values. In addition, occasional aggregates of two or more dimers with colocalized N-terminal portions were observed. Arrowheads mark positions of CK domains. Scale bars are 50 nm, ranges of color scale are 2.4 nm.
(EPS)

S3 Fig AFM imaging of multimeric VWF samples. Shown is an exemplary image of a wildtype VWF sample purified via centrifuge filtration only. Importantly, VWF molecules (indicated by circles) can clearly be distinguished from other molecules due to their large size and characteristic structure. The yellow circle highlights a rather large multimer of size *i* = 12. Scale bar is 200 nm, range of color scale is 2.4 nm.
(EPS)

S4 Fig Quantitative electrophoretic VWF multimer analysis. (A-E) Left: Luminescence intensity profiles (black lines) along cross-sections of the lanes of the blot shown in Fig 3 in the main text, and multi-Gaussian fits (red lines) to the profiles, for wtVWF (A) and mutants p.Cys1099Tyr (B), p.Cys2771Arg (C), p.Cys2773Arg (D), and p.Cys2811Ala (E). Right: Frequencies of multimers of size *i (i* = 1 corresponds to a dimer), determined from the areas of the fitted Gauss peaks after normalizing the area of each fitted Gauss peak by its corresponding multimer size *i*.
(EPS)

S1 Table Size distributions of wildtype and mutant VWF. Listed are the data presented in Fig 2 in the main text. Columns contain the numbers of observed molecules of size *i*, *(i* = 1 corresponds to a dimer) for the indicated VWF variants, with distinction between native (‘n’) and non-native (‘nn’) molecules. In the last column, the full results of the reopening simulation presented in Fig 2Eii are listed.
(XLSX)
